# Germination effects on the physicochemical properties and sensory profiles of velvet bean (*Mucuna pruriens*) and soybean tempe

**DOI:** 10.3389/fnut.2024.1383841

**Published:** 2024-04-16

**Authors:** Made Astawan, Ayu Putri Gitanjali Prayudani, Muhammad Haekal, Tutik Wresdiyati, Ratnaningsih Eko Sardjono

**Affiliations:** ^1^Department of Food Science and Technology, Faculty of Agricultural Engineering and Technology, IPB University, Bogor, Indonesia; ^2^School of Veterinary Medicine and Biomedicine Sciences, IPB University, Bogor, Indonesia; ^3^Department of Chemistry, Indonesia University of Education, Bandung, Indonesia

**Keywords:** CATA, germination, physicochemical, sensory profile, velvet bean tempe

## Abstract

**Introduction:**

Previous studies have shown that the velvet bean, an indigenous legume in Indonesia, possesses high protein content and bioactive compounds. However, the utilization of velvet beans in tempe production remains underexplored.

**Methods:**

This study aims to address this research gap by investigating the physicochemical properties and sensory profiles of tempe made from velvet beans, both individually and in combination with soybean. The study involved the production of tempe using germinated and non-germinated velvet bean, soybean, and a soy-velvet bean combination (61:39% ratio). Physicochemical analyses, including hardness, firmness, colour, antioxidant capacity, proximate, pH, and titratable acidity, were conducted. Hedonic rating and Check-All-That-Apply (CATA) tests were also performed to assess the sensory attributes of fresh and fried tempe.

**Results and discussion:**

Germination treatment of velvet bean resulted in tempe with reduced hardness, firmness, antioxidant capacity, and pH levels compared to non-germinated velvet bean tempe. However, velvet bean tempe exhibited a darker colour, higher antioxidant capacity, higher pH levels, and lower titratable acidity compared to soybean tempe and soy-velvet bean combination tempe. The protein content in velvet bean tempe was found to be below the required threshold of 15%. Hedonic rating tests revealed that fresh and fried velvet bean tempe received lower scores than other samples. CATA tests identified specific sensory attributes essential for fresh and fried tempe, including beany aroma, white colour, nutty aroma, golden brown colour, solid and crunchy texture, umami taste, and nutty aftertaste. These findings provide valuable insights into the potential applications of velvet beans in tempe production and emphasize the significance of considering germination as a factor affecting the quality and sensory attributes of tempe.

## 1 Introduction

Tempe is a traditional Indonesian food product that is made from the fermentation of soybean by *Rhizopus* spp. mold. Soy-based tempe contains relatively high protein content, that is 40% of dry basis (1). This causes tempe to be acknowledged as an affordable source of protein by Indonesian consumers (2). Tempe also contains various bioactive compounds, such as isoflavones which act as an antioxidant. The fermentation process converts glycone isoflavones (in soybean) into aglycone isoflavones (in tempe), which are easier to digest by the human body (3).

Indonesia is acknowledged as the biggest tempe producer in the world, even 70% (around 1.8 million tons) of total soybean in Indonesia are used for tempe production (4). In 2020, the tempe consumption in Indonesia managed to reach 7.29 kg/capita/year. According to data from the Indonesian Central Bureau of Statistics, soybean imports in Indonesia in 2021 reached 2.5 million tons, or equivalent to US$ 1.48 billion. Therefore, local commodity alternative aside from soybean is needed, with one of them being velvet bean (*Mucuna pruriens*), or known as *koro benguk* in Indonesian.

Velvet bean has a higher productivity compared to soybeans. The productivity of velvet bean bean is around 3–4 tons/ha, higher than the productivity of soybeans which is around 1–2 tons/ha (5). Velvet bean also has a relatively high protein content, which is 28.4–31.0% (5). Furthermore, velvet beans also contain active components such as L-3,4-dihydroxyphenylalanine (L-DOPA) that can alleviate the symptoms of Parkinson’s disease (6). As one of Indonesia’s local beans, velvet bean has the potential to be utilized as a raw material in tempe production. Several studies have used velvet bean as a raw material for tempe production. Rahayu et al. (5) found changes in velvet bean protein during fermentation, producing protein fragments with a molecular weight less than 25 kDa. Research by Fitriyah et al. (7) shows that velvet bean tempe flour has potential as an alternative source of protein and calcium. Recent study also demonstrate that feeding experimental rats with a combination of velvet bean and soybean tempe resulted in FCE, growth rate, and NPR values that are comparable to those of soybean (8). Nevertheless, the main weaknesses of velvet beans are the high content of antinutrient compounds (tannin, saponin, and cyanide acid/HCN) and hard texture. Therefore, innovation in the production process is needed to produce tempe that is safe to consume and can be accepted by consumers sensory-wise.

One of the innovations that can be done is through velvet bean seed germination before tempe processing. Germination process may enhance and increase the bioavailability of the nutritional profile (9). Germination treatment also can reduce anti-nutrients like phytates, tannins, and oxalic acid (10). The germination of velvet bean seeds has been proven to significantly reduce the HCN content from 19.88 to 2.87 mg/kg dry basis and increase the total phenolic content from 1.95 to 2.55 g GAE/100 g dry basis (11). In soy-based tempe, the germination of soybean seeds had proven to decrease the hardness value and firmness of the resulting tempe (12). Meanwhile, germination of velvet bean reduced antioxidant capacity, total phenol, and GABA content, but increased the protein content in the resulted velvet bean tempe (3).

The purpose of this study was to evaluate the physicochemical and sensory of tempe made from velvet bean seeds or their combination with soybean. The samples consisted of 100% velvet bean tempe (germinated and non-germinated), 100% soybean tempe, and a combination of soybean-velvet bean tempe with a ratio of 61:39% (w/w). The analyses consisted of physicochemical (proximate, antioxidant capacity, pH, titratable acidity/TA, colour, and texture) and organoleptic (hedonic rating test and check-all-that-apply/CATA test). Additionally, this study identified both the desirable and undesirable attributes of both fresh and fried velvet bean tempe, providing novel insights into its sensory properties.

## 2 Materials and methods

### 2.1 Chemicals and materials

The materials used in this study were Genetically Modified Organism (GMO) soybeans (Sb&B Food Inc., Casselton, United States), and velvet bean seeds purchased from PT. Nagari Bumi Asri (Yogyakarta, Indonesia) with a chemical composition that can be seen in our previous study (3), Raprima^®^ tempe starter (Bandung, Indonesia), and polypropylene plastic as tempe packaging. Moreover, the materials used for analysis consisted of reagents for proximate analysis, 1-diphenyl-2-picrylhydrazyl (DPPH), ascorbic acid, Folin–Ciocalteu, and gallic acid from Sigma-Aldrich (United States).

### 2.2 Tempe production process

The tempe production process was started with the sortation of velvet bean seeds, initial soaking for 24 h, initial boiling for 60 min, dehulling, second soaking for 24 h, washing, second boiling for 15 min, draining, inoculation, packaging, and fermentation. The production of germinated velvet bean tempe had similar steps to the non-germinated velvet bean tempe. The difference lies in the germination process after the initial soaking. The soaked velvet bean seeds were then placed in a tray covered with a damp cloth to be covered and moistened every 6 h to maintain the moisture of the seeds. The germination process lasted for approximately 24 h until a radicle with a length of 3–5 mm sprouted.

In the tempe production process for soybean-velvet bean treatment, there was a mixing soybean with velvet bean step before the inoculation process. The ratio of soybean-velvet bean (69:31%, w/w) was chosen based on formula optimization that had been done by the previous study by observing two primary parameters, such as hardness and protein content (13). While other treatments used 100% velvet bean (germinated and non-germinated), and 100% soybean as raw material for tempe.

### 2.3 Chemical analysis

The analysis conducted on fresh tempe consisted of proximate analysis (moisture, ash, protein, fat, and carbohydrate) and crude fiber with the AOAC method (14). Water and ash content were analysed using the gravimetric method (AOAC 925.09 and AOAC 923.03). Protein content was analysed using Kjeldahl method (AOAC 955.04D). Fat content was analysed using Soxhlet extraction method (AOAC 922.06). Meanwhile, carbohydrate was calculated using the difference of 100 subtracted by the total of water, ash, protein, and fat content. Crude fiber was analysed using the acid and base hydrolysis method (AOAC 962.09).

The pH analysis was done using pH meter (Orion Star A121, Thermo Scientific, United States) which had been calibrated at pH 4.0 and 7.0. Then, 10 grams of tempe was mashed in a mortar and the water was added slowly (1:5 b/v). Then, the pH sample was measured. The titratable acidity (TA) analysis was done by taking 10 mL of sample solution obtained from the pH analysis step then added with 3 drops of phenolphthalein indicator to be titrated with 0.1 N NaOH (15).

The antioxidant capacity analysis was conducted with DPPH assay on fresh tempe sample that was floured using fluidised bed dryer method (16). One gram of tempe flour sample was diluted in 10 mL methanol, then centrifuged at 4°C with the speed of 2000 x g for 45 min (Eppendorf 5810R). The analysis was done by collecting 1 mL of each supernatant (for soybean tempe sample), 0.5 mL (for soybean and velvet bean tempe mixture sample), 0.25 mL (for velvet bean tempe sample) and then added with methanol until the volume reached 1 mL. The sample was then added with 3 mL of 0.5 mM DPPH reagent and vortexed. Next, the sample was kept in a dark room for 30 min before measuring the absorbance (A_1_) at 517 nm wavelength. The blank sample without sample addition was also added to determine the absorbance (A_0_). The standard curve was created from several ascorbic acid concentrations, namely 0.02 mg/mL, 0.04 mg/mL, 0.06 mg/mL, 0.08 mg/mL, 0.10 mg/mL, and 0.12 mg/mL. The tempe antioxidant capacity was determined by dividing the percentage of sample inhibition and ascorbic acid using formula as following:


$$\%\text{inhibition}=\frac{\left(A_{0}-A_{1}\right)}{A_{0}}\times 100$$

### 2.4 Physical analysis

Physical analysis on fresh tempe consisted of hardness and firmness with texture analyser TA-XT2i (Stable Microsystem, United Kingdom) (17). Tempe sample with dimension of 1.5 × 1.5 × 1.5 cm was analysed using TA-43 probe (knife-like) with the speed of 1.5 mm/s and distance of 30 mm. The peak and area beneath the curve were reported each as hardness and firmness/firmness.

The colour analysis was done in the surface and inner parts of tempe using Minolta chromameter CR 310 (18) as a representation of hyphae and beans. The device was calibrated before analysis with a white plate. The measurement was done by shooting the device on a flat surface, with the results reported as L, a*, and b* scale values.

### 2.5 Sensory evaluation

The sensory analysis on fresh and fried tempe was done using check-all-that-apply (CATA) method and hedonic rating on parameters such as colour, texture, aroma, overall, and taste (only on fried tempe). Before the sensory test, a focus group discussion (FGD) was held on 10 untrained panelists. The FGD was done to determine the attributes on fresh and fried tempe which would be assessed by the panelists (19).

The sample preparation was started by cutting the tempe so it would form bar with dimension of 4 × 3 × 1 cm. The cut tempe was fried with cooking oil at 170°C for 15 min. The involved panelists were 74 untrained panelists (23 males and 51 females, aged between 18 to 22 years). On CATA test, the panelists would choose several attributes in each parameter that had been previously discussed on fresh and fried tempe sample without comparing one to another. Then, in hedonic rating test, the panelists would give their assessment in each parameter on scale 1–7, such as highly dislike (1), dislike (2), slightly dislike (3), neutral (4), slightly like (5), like (6), and highly like (7).

### 2.6 Data analysis

The study was conducted with a completely randomized design. The data obtained was processed using SPSS software (20). ANOVA analysis was used to analyse physicochemical properties and hedonic rating test. If the results were significantly different at a 5% significance level, it was followed by post-hoc Duncan Multiple Rank Test (DMRT). CATA analysis was conducted to analyse CATA test and was carried out using XLSTAT application with the CATA data analysis tool (21).

## 3 Results and discussion

### 3.1 Physical properties

Colour analysis on both surface and internal part of tempe was carried out using chromameter device. The result of colour analysis on the tempe surface is shown on Table 1 which showed that there was significant difference on lightness parameter (L*) and b* parameter. Tempe from velvet bean seeds had lower brightness level compared to soybean tempe and soybean-velvet bean tempe. On b* parameter, the analysis result showed that soybean tempe and soybean-velvet bean tempe had similar and significantly more yellowish colour compared to velvet bean tempe.

**Table 1:** Physical properties of various tempe.

Parameter	Type of tempe				
	GV	NGV	S-GV	S-NGV	S
Colour on the surface					
L*	81.99 ± 1.55^a^	81.51 ± 0.89^a^	85.60 ± 0.93^b^	85.04 ± 0.91^b^	85.54 ± 0.18^b^
a*	0.14 ± 0.11	0.41 ± 0.25	0.47 ± 0.01	0.44 ± 0.03	0.56 ± 0.11
b*	5.83 ± 0.56^a^	6.73 ± 0.85^a^	9.42 ± 0.19^b^	9.40 ± 0.16^b^	9.34 ± 0.06^b^
Hue	88.44 ± 1.40	86.18 ± 2.51	87.16 ± 0.06	87.30 ± 0.15	86.58 ± 0.70
Colour on the inner part					
L*	62.51 ± 0.45^a^	63.42 ± 1.09^a^	70.25 ± 0.53^b^	75.56 ± 1,27^c^	78.44 ± 1.05^d^
a*	1.88 ± 0.48	1.98 ± 0.11	1.14 ± 0.07	1.06 ± 0.52	0.95 ± 0.16
b*	8.36 ± 0.18^ab^	7.11 ± 0.27^a^	13.10 ± 3.10^bc^	17.15 ± 2.77^cd^	20.71 ± 0.37^d^
Hue	77.37 ± 2.87^a^	74.25 ± 1.54^a^	84.23 ± 2.16^b^	86.09 ± 2.53^b^	87.35 ± 0.48^b^
Hardness (N)	14.36 ± 1.58^ab^	18.21 ± 1.93^b^	11.58 ± 1.99^a^	12.11 ± 2.16^a^	10.18 ± 0.004^a^
Firmness (N.s)	87.20 ± 8.18^a^	110.25 ± 1.02^b^	83.99 ± 12.62^a^	83.99 ± 5.95^a^	73.47 ± 5.07^a^

*Results are expressed as means of four determinations ± standard deviation.

GV, Germinated velvet bean; NGV, Non-germinated velvet bean; S-GV, Soy-germinated velvet bean; S-NGV, Soy-nongerminated velvet bean; S, Soybean.

Values in the same row with the same letter in superscript are not statistically different (*p* < 0.05).

The colour analysis of tempe internal part was also shown on Table 1 which showed there was significant differences (*p* < 0.05) on lightness (L*), b*, and hue parameters. On lightness parameter, velvet bean tempe had lower lightness compared to soybean tempe and soybean-velvet bean tempe. Soybean and soybean-velvet bean tempe were more yellow compared to velvet bean tempe, indicated by the lower b* value of velvet bean tempe compared to other treatment.

The hue scale showed the value that represents the combination of colours in a sample. Soybean tempe and soybean-velvet bean tempe had higher hue value compared to velvet bean tempe. It showed that soybean tempe and soybean-velvet bean tempe had a more yellow greenish colour compared to 100% velvet bean tempe. The colour analysis was done according to the previous study in velvet bean flour tempe sample. The study mentioned that velvet bean tempe flour had darker colour compared to soybean tempe flour (22).

**Figure 1:**
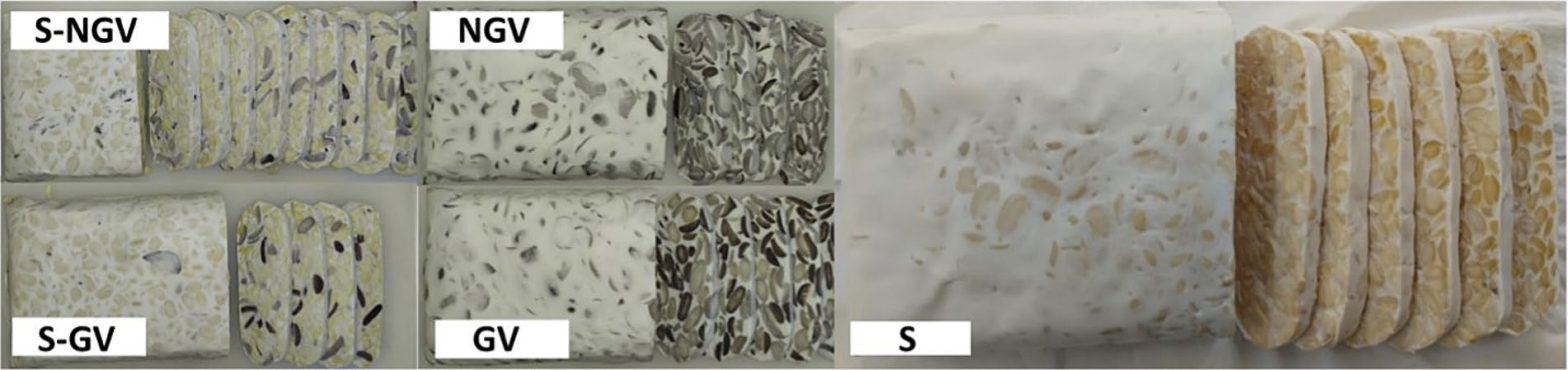
Visual tempe made of: GV, Germinated Velvet Bean; NGV, Non-Germinated Velvet Bean; S-GV, Soy-Germinated Velvet Bean Combination; S-NGV, Soy-Non Germinated Velvet Bean; S, Soybean.

Colour analysis with chromameter showed similar result with visual observation. On Figure 1, germinated velvet bean (GV) tempe and non-germinated velvet bean (NGV) tempe both had darker appearance compared to germinated soybean-velvet bean (S-GV) tempe as well as non-germinated soybean-velvet bean tempe (S-NGV), and significantly different than tempe made from soybean. Germination treatment on both velvet beans and soybeans showed a decrease in lightness (L*) compared to non-germination treatment. The presence of polyphenol compounds in the seeds, along with the activation of polyphenol oxidase enzymes during the germination process, is likely responsible for this outcome, as explained by Vadivel and Biesalski (23). These enzymes facilitate the conversion of polyphenolic compounds into quinone group compounds, contributing to the seeds’ dark colour (24). In addition, according to Astawan et al. (3), the reduced lightness observed in germinated velvet bean and soybean tempe is caused by the high concentration of amino acids in the seeds that result from the germination process. When the seeds undergo heating or boiling during tempe production, these amino acids undergo non-enzymatic browning, such as the Maillard reaction. Additionally, Oskaybaş-Emlek et al. (25) reported a decrease in lightness in germinated lentil flour due to an increase in phenolic compounds and protein content. Another explanation by Glagoleva et al. (26) stated that melanin, anthocyanins, and other phenolic compounds are pigments found in seeds, and their activity can be increased during germination, thus affecting the darker colour of the tempe produced.

Table 1 also showed the firmness and hardness of fresh tempe. The analysis showed that the germination of velvet bean seeds was able to reduce the hardness and firmness of tempe. One of the main weaknesses possessed by velvet bean seeds is its tough texture. The previous study also showed that velvet bean seeds had higher hardness than soybeans (7). The process of velvet bean germination and combining it with soybean was expected to produce tempe that has similar texture with soybean tempe.

The results of the analysis demonstrated that the NGV sample exhibited the highest firmness and hardness. In contrast, the 24-h germination process was found to decrease hardness and firmness of the GV and S-GV samples. This phenomenon is consistent with the findings of previous studies that have reported a reduction in tempe firmness and hardness due to the length of germination and fermentation (12). In soy-based yogurt, the hardness of the product decreased with the growth of hypocotyl resulting from germination (27). Moreover, research reported by Zinia et al. (28) has demonstrated that as the soybean germination process increases, the hardness of the resulting tofu decreases. This phenomenon is attributed to the hydrolysis activity of components in the seeds during the germination process (29). Germination triggers proteolytic enzymes that break down protein aggregation and molecular interactions, leading to a looser protein morphological structure (30). Additionally, germination also increases the porosity of soybean cell walls, decreases the density of intracellular tissues, and alters the macrostructure of proteins (31). Furthermore, germination-induced enzymatic activities lead to the breakdown of starch into simpler sugar, which affect the nutritional composition and texture by softening the seeds, shown by an increased pores of lentil starch granules due to partial hydrolysis during germination (25). Therefore, germination-induced enzymatic activities involved in texture modification, making the resulting tempe softer and less firm compared to non-germinated samples.

### 3.2 Chemical properties

The comparison of proximate composition of various tempe is as shown in Table 2. Tempe from velvet bean had significantly lower ash content (*p* < 0.05) compared with soybean tempe and soybean-velvet bean tempe. In addition, the velvet bean germination process also resulted in tempe with lower ash content than similar tempe with non-germination treatment. The ash content is influenced by the amount of mineral within the food material (32). Germination and boiling time contribute the mineral content of tempe. The germination process resulted in velvet bean seeds being exposed to water continuously which would result in minerals being dissolved into the water (33). The boiling time tended to reduce the ash content, although it was statistically insignificant (34). Both soybean and soybean-velvet bean tempe had significantly higher protein content than velvet bean tempe. On the contrary, the carbohydrate content of velvet bean tempe was significantly higher than soybean and soybean-velvet bean tempe. Velvet bean tempe had lower crude fiber content compared with soybean and soybean-velvet bean tempe. This was due to the beans used as tempe raw material had different initial nutritional composition.

**Table 2:** Proximate composition of various tempe per 100 g.

Parameter	Type of tempe					INS
	GV	NGV	S-GV	S-NGV	S	
Moisture	60.05 ± 1.15^a^	61.97 ± 0.98^a^	61.83 ± 0.20^a^	62.42 ± 0.11^a^	61.17 ± 0.27^a^	Max 65.0
Ash	0.47 ± 0.01^a^	0.50 ± 0.06^a^	0.75 ± 0.08^b^	0.91 ± 0.02^c^	1.02 ± 0.10^c^	–
Protein	12.68 ± 0.05^a^	13.34 ± 0.03^b^	16.35 ± 0.41^c^	16.57 ± 0.64^c^	20.08 ± 0.24^d^	Min 15.0
Fat	1.84 ± 0.12^b^	1.08 ± 0.06^a^	3.75 ± 0.08^c^	4.38 ± 0.25^d^	8.47 ± 0.13^e^	Min 7.0
Carbohydrate	24.96 ± 1.31^c^	23.11 ± 0.71^c^	17.32 ± 0.22^b^	15.73 ± 0.98^b^	9.27 ± 0.74^a^	–
Crude fiber	0.53 ± 0.07^a^	0.90 ± 0.09^b^	2.26 ± 0.45^d^	2.34 ± 0.04^d^	1.47 ± 0.04^c^	Max 2.5

*Results are presented on wet basis. Values are means of four determinations ± standard deviation.

INS, Indonesian National Standard; GV, Germinated velvet bean; NGV, Non-germinated velvet bean; S-GV, Soy-germinated velvet bean; S-NGV, Soy-nongerminated velvet bean; S, Soybean.

Values in the same row with the same letter in superscript are not statistically different (*p* < 0.05).

Table 2 also shows the proximate composition of tempe that was compared with the current Indonesian National standard (SNI). All tempe types had similar water content, which was 60–62%, thus it fulfills the SNI requirements, which is below 65%. Velvet bean tempe had lower protein content than the SNI requirement, which is 15% minimum. The mixture of soybean and velvet bean tempe with ratio of 69:31% (w/w) was able to produce tempe with protein content of 16%. The highest protein content of 20% was possessed by soybean tempe. Protein content is one of the most crucial parameters in tempe because tempe is known as a source of protein (35). Velvet bean tempe also had lower fat content than soybean-velvet bean tempe and soybean tempe. This was due to different fat content in the initial raw materials. Velvet bean had 4% fat content, whereas soybean had 20.58% fat content (11). This resulted in only soybean tempe which fulfilled the minimum fat content requirement, particularly 7.0%.

Table 3 shows the pH value, titratable acidity (TA), and antioxidant capacity of five types of tempe. Velvet bean tempe had higher pH than soybean and soybean-velvet bean tempe. Soybean tempe had the lower pH. On the contrary, the TA analysis showed that velvet bean tempe had the lowest value than soybean and soybean-velvet bean tempe. The germination treatment on velvet bean seeds affected the pH decrease and TA value increase. In other study, it was mentioned that germination treatment on lupin (*Lupin albus*), chickpeas (*Cicer aretinium* L.), soybean (*Glycine max* L.), and lentils (*Lens culinaris* Merr.) decreased the pH value or increase the bean acidity along with the length of the germination duration (36). This was caused by the germination process which caused the conversions of carbohydrates into organic acids, protein into free amino acids, and fat into fatty acids. Similar explanation was also mentioned in the precious study which stated that soybean germination resulted the carbohydrate conversion into several organic acids, namely malonic acid, fumaric acid, malic acid, citric acid, and glucaric acid (37). Similar result was also shown by Jiang et al. (38) which explained that soymilk produced from germinated soybean had lower pH compared with soymilk from non-germinated soybean.

**Table 3:** Analysis results for pH, titratable acidity, and antioxidant capacity of various tempe.

Type of tempe	pH	Titratable acidity	Antioxidant capacity (mg AEAC/100 g tempe flour)
GV	6.32 ± 0.03^cd^	2.77 ± 0.51^a^	102.37 ± 12.72^b^
NGV	6.52 ± 0.08^d^	2.53 ± 0.17^a^	289.56 ± 35.76^d^
S-GV	5.64 ± 0.11^b^	4.34 ± 0.34^b^	57.16 ± 6.25^a^
S-NGV	6.06 ± 0.20^c^	3.37 ± 0.00^b^	190.40 ± 5.95^c^
S	5.02 ± 0.00^a^	3.98 ± 0.52^b^	20.87 ± 0.91^a^

*Results are expressed as means of four determinations ± standard deviations.

GV, Germinated velvet bean; NGV, Non-germinated velvet bean; S-GV, Soy-germinated velvet bean; S-NGV, Soy-nongerminated velvet bean; S, Soybean.

Values in the same column with the same letter in superscript are not statistically different (*p* < 0.05).

Velvet bean tempe had higher antioxidant capacity than soybean tempe. Non-germinated velvet bean tempe had higher antioxidant capacity than germinated velvet bean tempe. Similar result was found on N-SGV sample which had higher antioxidant capacity than S-GB sample. The germination treatment on velvet bean could reduce its antioxidant capacity. However, the antioxidant capacity value of germinated velvet bean tempe was higher than soybean tempe. Phenolic compounds are the most important antioxidant in plants (39). The phenolic compounds in velvet bean seeds extract reached 6.48 g/100 g (40). Other study also mentioned that velvet bean seeds contained 5.65% phenolic compounds (41). The decline of antioxidant capacity in germinated velvet bean tempe was caused by the decline of phenolic compounds during the germination process. In the previous study, germination treatment on velvet bean was found to reduce the total phenolic content and various antinutrient compounds such as tannin, phytic acid, and trypsin inhibitor (42).

### 3.3 Sensory properties

Sensory evaluation is crucial in determining the acceptability and preference of food products by consumers. In this study, both the check-all-that-apply (CATA) and hedonic rating tests were conducted to gain insights into the sensory attributes preferred by the panelists for fresh and fried tempe. Based on the focus group discussion (FGD) result, 16 potential sensory attributes were found on fresh tempe sample, and 29 potential sensory attributes were found on fried tempe sample which can be seen in Table 4. The Cochran’s Q test provides significant information regarding the difference of each attribute on samples that are tested on the panelists (43). If an attribute has *p*-value >0.05, there are no significant differences found between the samples. Five fresh tempe samples were significantly different (*p* < 0.05) on all attributes, except for mushroom aroma, nutty aroma, green colour, and fibrous texture.

**Table 4:** Sensory attributes of fresh and fried tempe.

Type of tempe	Aroma	Texture	Colour	Flavor	Mouthfeel	Aftertaste
Fresh tempe	Beany	Soft	White	–	–	–
	Mushroom	Solid	Yellow			
	Nutty	Hard	Grey			
	Fermented/sour	Brittle	Brown			
	Alcoholic	Springy	Black			
		Fibrous	Green			
Fried tempe	Beany	Soft	White	Salty	Oily	Bitter
	Mushroom	Hard	Yellow	Savory	Fatty	Sour
	Nutty	Solid	Grey	Sour	Grittiness	Astringent
	Fermented/sour	Brittle	Black	Bitter		Nutty
	Alcoholic	Crunchy	Golden brown	Sweet		
	Burned					
	Rancid					

**Figure 2:**
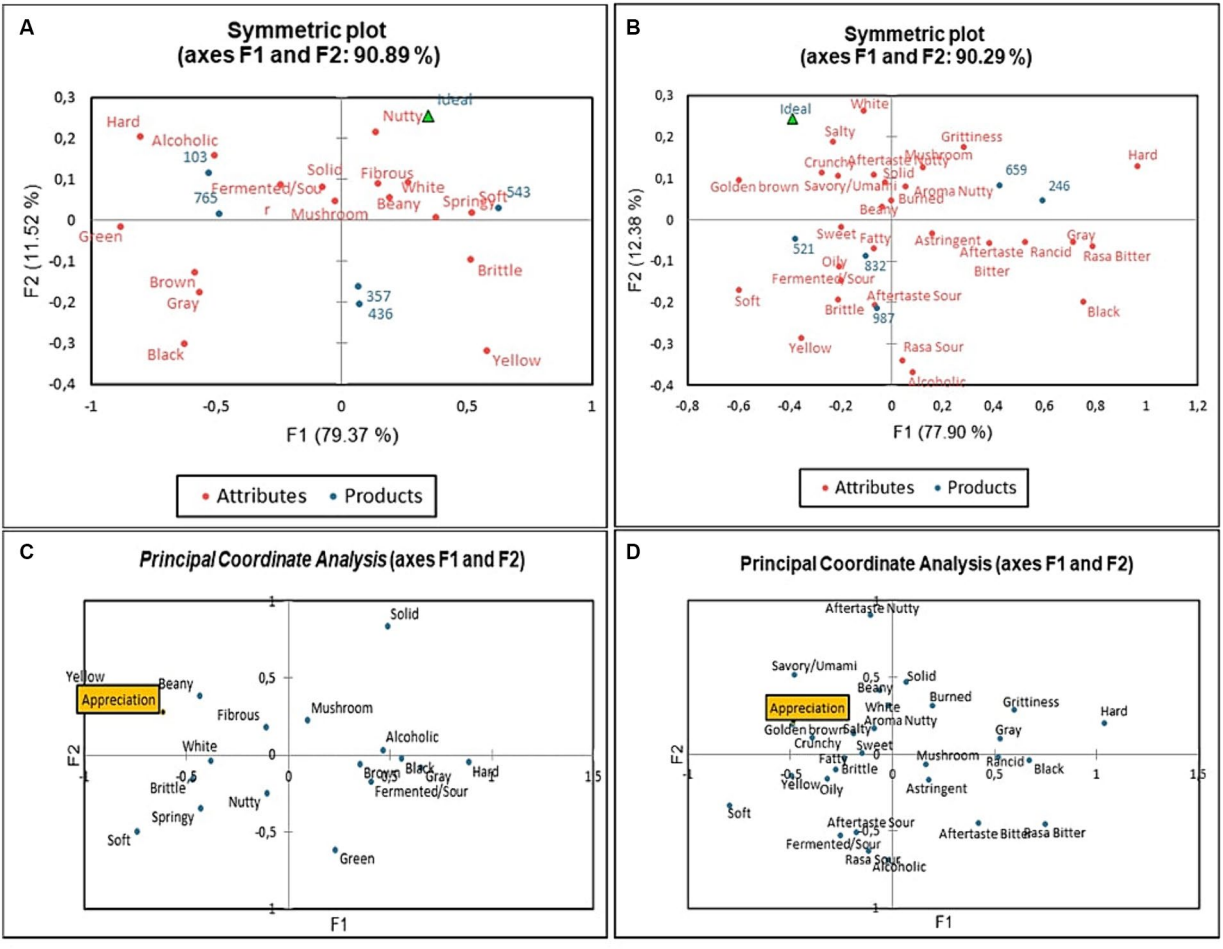
Biplot graph of principal component analysis from: **(A)** Samples and ideal fresh tempe sensory profile: Code 103 (GV) = Germinated Velvet Bean Tempe, 765 (NGV) = Non Germinated Velvet Bean Tempe, 357 (S-GV) = Soy-Germinated Velvet Bean Tempe Combination, 436 (S-NGV) = Soy-Non Germinated Velvet Bean Tempe, 543 (S) = Soybean Tempe. **(B)** Samples and ideal fried tempe sensory profile: Code 659 (GV) = Germinated Velvet Bean Tempe, 246 (NGV) = Non Germinated Velvet Bean Tempe, 832 (S-GV) = Soy-Germinated Velvet Bean Tempe, 987 (S-NGV) = Soy-Non Germinated Velvet Bean Tempe, 521 (S) = Soybean Tempe. **(C)** Sensory attributes of fresh tempe with panelist preferences. **(D)** Sensory attributes of fried tempe with panelist preferences.

The profile of fresh and fried tempe presented to the panelists is shown on biplot map (Figure 2). In Figure 2A, fresh tempe is ideal if it possesses a nutty aroma, beany aroma, fibrous texture, springy texture, soft texture, and white colour attributes. On soybean tempe sample (code 543), had the closest profile to ideal fresh tempe according to the panelists. If compared with velvet bean tempe (codes 103 and 765), it was found that soybean-velvet bean tempe (codes 357 and 436) had sensory attributes that tended to resemble soybean tempe, but the profiles were in a different quadrant.

Based on Cochran’s Q test analysis, all five fried tempe samples were significantly different (*p* < 0.05) in fermented aroma, yellow colour, grey colour, black colour, golden brown colour, soft texture, hard texture, brittle texture, crunchy texture, savory taste, salty taste, sour taste, bitter taste, oily mouthfeel, grittiness mouthfeel, bitter aftertaste, and sour aftertaste attributes. The correspondence analysis represents the ideal sensory profile of fried tempe according to the panelists. In Figure 2B, the ideal fried tempe according to the panelists had dominant attributes such as golden-brown colour, white colour, salty taste, umami taste, crunchy texture, solid texture, nutty aftertaste, and beany aroma. The correspondence analysis also showed that soybean-velvet bean tempe (codes 832 and 987) possessed a sensory profile that was similar to fried soybean tempe (code 521).

The principal coordinate analysis is a graph that shows the relationship between attributes in the samples and overall product liking (43), which identified through the CATA test and hedonic rating test. An attribute is considered to contribute to increasing the consumer liking of a product if the attribute is close to the appreciation point (44). In Figure 2C, it is known that the panelists’ preference for fresh tempe increased if it had a yellow colour, beany aroma, and fibrous texture attributes. Meanwhile, in Figure 2D, the result of the principal coordinate analysis on fresh tempe showed that the golden-brown colour, savory/umami taste, salty taste, crunchy texture, beany aroma, nutty aroma, and nutty aftertaste attributes were several attributes in fried tempe which were favored by the panelists.

Penalty analysis can be used to provide information regarding the attributes that can increase and decrease the panelists’ liking (45). In penalty data analysis, there are several terms, namely: P (No) | (Yes), P (Yes) | (Yes), P (No) | (No), and P (Yes) | (No). To summarize, the penalty analysis can be divided into several aspects, such as “must have,” “nice to have,” and “must not have” (43). The “must-have” sensory attribute is an attribute that does not present in a product presented to the panelists, but the attribute is desired by the panelists because it is considered as the attribute of an ideal product. The determination of a certain attribute to be categorised as “must-have” is based on P (No) | (Yes), P (Yes) | (Yes) conditions. An attribute has the potential to be categorised as a “must-have” if it has positive means drop value and P (No) | (Yes) condition of more than 20% (46).

**Figure 3:**
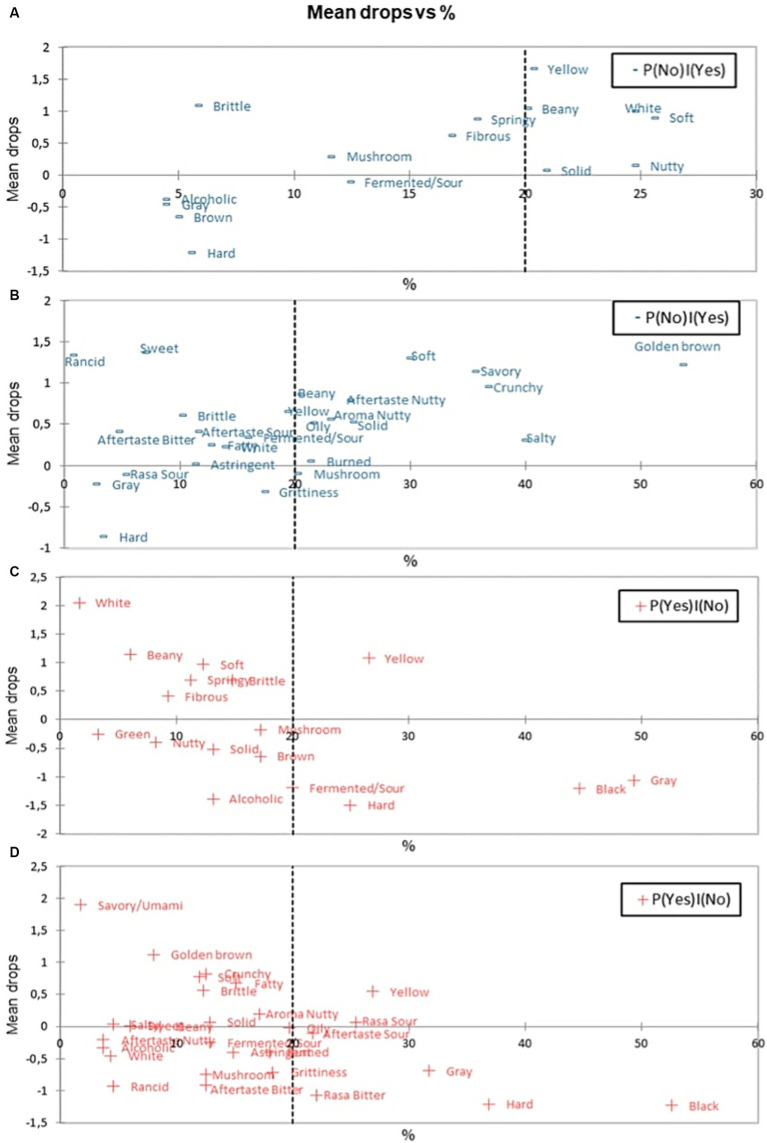
Mapping plot of sensory attributes on various samples: **(A)** “Must have” on fresh tempe. **(B)** “Must have” on fried tempe. **(C)** “Must not have” on fresh tempe. **(D)** “Must not have” on fried tempe.

The means drop chart result versus the P (No) | (Yes) fresh and fried tempe samples can be seen in Figure 3. On fresh tempe, beany aroma and white colour were included as “must have” attributes. On fried tempe, beany aroma, nutty aroma, golden brown colour, solid texture, crunchy texture, umami/savory taste, and nutty aftertaste were categorised as “must have” attributes. This analysis validated previous findings as optimal sensory profile of tempe preferred by consumers.

“Nice to have” and “must not have” attributes are attributes that are found in the product by the panelists, but not found in the ideal product. The presence of “nice to have” attribute will increase the panelist liking on the product, while the “must not have” attribute will reduce the panelist’s liking of the product (47). The determination of “nice to have” and “must not have” attributes were based on P(No) | (No) dan P (Yes) | (No) conditions that were presented in Figures 3C,D charts. An attribute is defined as “nice to have” if it has more than 20% P (Yes) | (No) condition, positive means drop, and *p*-value <5%. In fresh tempe and fried tempe, the attribute that was categorised as “nice to have” was a yellow colour attribute, because the attribute had P (Yes) | (No) above 20%, positive means drop, and *p*-value <5%.

An attribute is categorised as “must not have” if the attribute has more than 20% *P* (Yes) | (No) condition, negative means drop, and *p*-value <5% (46). On the fresh tempe sample, the attributes which were categorised as “must not have” attributes were sour/fermented aroma, grey colour, black colour, and hard texture attributes. On fried tempe, the attributes which were classified as “must not have” attributes were grey colour, black colour, bitter taste, and hard texture.

The hedonic rating test was carried out to determine the panelists’ or consumers’ preference on a food product (48). The hedonic rating test for fresh and fried tempe was done on all five samples and presented in Table 5. The hedonic rating test result of fresh tempe showed that all five samples were significantly different (*p* < 0.05) on aroma, colour, overall, texture, and appearance parameters. For fried tempe, all five samples were significantly different (*p* < 0.05) on all parameters; aroma, colour, taste, overall, mouthfeel, aftertaste, texture, and appearance. To conclude, velvet bean tempe had a lower preference level compared with fried soybean-velvet bean tempe and soybean tempe.

**Table 5:** Hedonic rating test of various tempe.

Parameter	Type of tempe				
	Germ Velvet	Nongerm Velvet	Soy-Germ Velvet	Soy-Nongerm Velvet	Soybean
***Fresh tempe***					
Aroma	3.75^a^	4.14^a^	5.00^b^	5.15^bc^	5.58^c^
Colour	2.82^a^	3.10^a^	4.63^b^	5.00^b^	6.38^c^
*Overall*	3.44^a^	3.70^a^	5.01^b^	5.15^b^	6.01^c^
Texture	3.89^a^	4.03^a^	5.07^b^	5.34^bc^	5.74^c^
Appearance	3.01^a^	3.21^a^	4.79^b^	4.92^b^	6.26^c^
***Fried tempe***					
Aroma	4.67^a^	4.59^a^	4.96^ab^	5.24^b^	5.83^c^
Colour	2.34^a^	2.69^a^	4.71^b^	5.10^c^	6.41^d^
Taste	3.49^a^	4.01^b^	4.26^b^	4.81^c^	5.50^d^
*Overall*	3.30^a^	3.89^b^	4.34^c^	4.83^d^	5.77^e^
Mouthfeel	3.54^a^	4.10^b^	4.37^b^	4.59^b^	5.53^c^
*Aftertaste*	4.00^a^	4.23^a^	4.10^a^	5.11^b^	5.40^b^
Texture	2.90^a^	3.79^b^	4.59^c^	5.11^d^	5.83^e^
Appearance	2.36^a^	2.81^b^	4.49^c^	4.99^d^	6.19^e^

*Results are expressed as means of 70 determinations. Values in the same row with the same letter in superscript are not statistically different (*p* < 0.05).

## 4 Conclusion

Velvet bean tempe had darker colour, hardness, and higher firmness characteristics than soybean and soybean-velvet bean tempe. The germination process of velvet bean was able to deduce the hardness and tempe firmness. The proximate analysis showed that velvet bean tempe had a protein content below the necessary threshold. Velvet bean tempe had higher pH, lower TA (titratable acidity), and higher antioxidant activity than soybean and soybean-velvet bean tempe. The germination of velvet bean seeds affected the resulted tempe chemical properties. The hedonic rating test showed lower preferences on velvet bean tempe compared with soybean and soybean-velvet bean tempe. The CATA test identified both desired and undesired sensory attributes of fresh and fried tempe.

## Data availability statement

The original contributions presented in the study are included in the article/supplementary material, further inquiries can be directed to the corresponding author.

## Ethics statement

The studies involving humans were approved by Human Research Ethics Committee of the Bogor Agricultural Research University. The studies were conducted in accordance with the local legislation and institutional requirements. The participants provided their written informed consent to participate in this study.

## Author contributions

MA: Conceptualization, Funding acquisition, Supervision, Validation, Writing – review & editing, Methodology. AP: Writing – original draft, Writing – review & editing, Project administration. MH: Formal analysis, Investigation, Visualization, Writing – original draft, Software. TW: Conceptualization, Resources, Supervision, Writing – review & editing. RS: Supervision, Validation, Writing – review & editing.
